# Reply: Cisplatin-Associated Aortic Thrombosis: A Review of Cases Reported to the FDA Adverse Event Reporting System

**DOI:** 10.1016/j.jaccao.2021.01.005

**Published:** 2021-03-16

**Authors:** Alan C. Cameron, Karla B. Neves, Jeff White, Rhian M. Touyz, Ninian N. Lang

We are grateful to Dr. Nguyen and colleagues for their interest in our recent study (1), published in *JACC: CardioOncology,* that assessed the vascular effects of cisplatin-based chemotherapy in patients with testicular cancer. We read with interest their work that highlights 37 cases of aortic thrombosis associated with cisplatin in the U.S. Food and Drug Administration (FDA) Adverse Event Reporting System and in medical publications. Their data are important in further demonstrating the potential for these clinically serious adverse thrombotic effects of cisplatin-based chemotherapy. In that study, of the 37 patients studied, aortic thrombosis was associated with a serious clinical outcome in all patients, with 1 patient requiring limb amputation. There were also 4 deaths. The total number of patients treated with cisplatin during the period studied is not available from this adverse event reporting system. However, the data clearly highlight that the beneficial anticancer effects of cisplatin-based chemotherapy (2,3) can come at the cost of an increased risk of serious and potentially fatal adverse cardiovascular effects.

It is vital that we continue to improve our understanding of the mechanisms underlying this arterial prothrombotic effect of cisplatin. Our own studies demonstrated that acute endothelial dysfunction may be implicated, with a marked endothelial toxic effect in the immediate periexposure period (1). Controlled trials of preventive therapies are lacking and should examine the effects of antithrombotic agents, antiplatelet drugs, or statins. Prospective risk stratification is also a challenge, but close attention should be paid to those patients with pre-existing cardiovascular risk factors. Cigarette smoking was common in patients with aortic thrombus, and this is strongly associated with endothelial dysfunction and impaired endogenous fibrinolysis (4). The combination of cigarette smoking and cisplatin exposure may be particularly potent in the propensity for thrombogenesis and propagation. We must ensure that the impressive anticancer effects of cisplatin do not come at a potentially unacceptable cardiovascular cost. A collaborative approach among cardiologists, oncologists, and primary care physicians should be encouraged.
